# Machine-Learning-Assisted Cyclostationary Spectral Analysis for Joint Signal Classification and Jammer Detection at the Physical Layer of Cognitive Radio

**DOI:** 10.3390/s23167144

**Published:** 2023-08-12

**Authors:** Tassadaq Nawaz, Ali Alzahrani

**Affiliations:** Department of Computer Engineering, King Faisal University, Al-Ahsa 31982, Saudi Arabia; aalzahrani@kfu.edu.sa

**Keywords:** cognitive radios, signals classifications, stealthy jammer, cyclostationary spectral analysis, artificial neural networks

## Abstract

Cognitive radio technology was introduced as a possible solution for spectrum scarcity by exploiting dynamic spectrum access. In the last two decades, most researchers focused on enabling cognitive radios for managing the spectrum. However, due to their intelligent nature, cognitive radios can scan the radio frequency environment and change their transmission parameters accordingly on-the-fly. Such capabilities make it suitable for the design of both advanced jamming and anti-jamming systems. In this context, our work presents a novel, robust algorithm for spectrum characterisation in wideband radios. The proposed algorithm considers that a wideband spectrum is sensed by a cognitive radio terminal. The wideband is constituted of different narrowband signals that could either be licit signals or signals jammed by stealthy jammers. Cyclostationary feature detection is adopted to measure the spectral correlation density function of each narrowband signal. Then, cyclic and angular frequency profiles are obtained from the spectral correlation density function, concatenated, and used as the feature sets for the artificial neural network, which characterise each narrowband signal as a licit signal with a particular modulation scheme or a signal jammed by a specific stealthy jammer. The algorithm is tested under both multi-tone and modulated stealthy jamming attacks. Results show that the classification accuracy of our novel algorithm is superior when compared with recently proposed signal classifications and jamming detection algorithms. The applications of the algorithm can be found in both commercial and military communication systems.

## 1. Introduction

Cognitive Radio (CR) emerged as a result of recent breakthroughs in Software Defined Radios (SDR) and Machine Learning (ML), as well as neuroscience findings [1,2]. Due to Dynamic Spectrum Access (DSA) capability, CR technology has the potential to address the problems of the wireless spectrum shortage and inefficient spectrum utilisation [3,4]. In both TV white space (TVWS) CR networks [5,6,7,8,9] and 5G technology [10], DSA plays a vital role.

In CR networks, the Secondary Users (SUs) can use the radio spectrum with different intentions, which means the licit user uses the radio spectrum in a manner to remain compliant with the security needs of licensed Primary Users (PUs) and avoid interfering with other users, whereas malicious users transmit signals with the motive of interfering with or jamming the communications of the targeted radio system. Due to their broadcast nature, radio communications are susceptible to external attacks launched by malicious users. The physical layer (PHY-layer) is particularly exposed to radio frequency (RF) jamming attacks. RF jamming and anti-jamming are well-known in conventional radio communication systems. However, significant advances have been made in the last two decades in CR technology that allow the design and deployment of advanced intelligent jamming [11] and anti-jamming [12,13] systems.

The need for measurable communications security in the Internet-of-Things (IoT) and Cyber-Physical Systems (CPSs) frameworks [14,15,16] has recently necessitated the establishment of an appropriate paradigm. SHIELD, which comprises the methods and techniques for designing secure embedded systems [17], is an important step taken in this direction. TVWS research [18] has also tackled the problem of illegitimate user detection.

CR networks allow the devices to sense the neighbouring radio environments, decide about the occupation of channels, and reconfigure the transmission parameters to achieve the required quality of service. Spectrum sensing is embedded in modern anti-jamming systems and plays a key role in the detection and identification of interfering and jamming entities [19]. Furthermore, it is used to record a history of the malicious user’s activities to design more efficient anti-jamming strategies, e.g., in frequency hopping spread spectrum (FHSS) systems it can be used to change the hopping sequence in order to avoid the bands jammed by malicious users [20].

A number of sensing methods were introduced over the last decade, which include energy detector (ED) [21], cyclostationary feature detector (CFD) [22], and matched filter detector (MFD) [23]. ED has a reasonably simple implementation and does not need any prior information about the signal. It is also included in the IEEE 802.22 standard for spectrum sensing. However, the detection performance of ED is significantly degraded in low signal-to-noise (SNR) radio environments. The MFD is the optimal detector but needs thorough prior information on the PU signal. Therefore, a receiver with a dedicated architecture is needed for the PU signal, which makes it impossible to consider in most practical sensing scenarios. On the other hand, in most practical scenarios, CR devices need to detect low-power signals; therefore, a new sensing technique—namely, CFD—is presented in the literature. The CFD technique has the capability to distinguish licit signals from noise and interference in low SNR conditions with high accuracy, which are attained at the expanse of high computational complexity. CFD relies on the cyclostationary features of modulated communication signals, such as modulation rate and carrier frequency, to distinguish between signals. The CFD sensing methods compute the spectral correlation function (SCF) [24,25,26] of signals, since noise is a stationary process and has no spectral components at harmonics that make it suitable to be used in low SNR environments. SCF has been employed as a reliable method for signal categorisation [27,28,29] since it produces a unique pattern for each signal. Therefore, it can also be used as a reliable tool to distinguish between legitimate signals and jamming waveforms. In [30], the authors compared the most widely used sensing methods in terms of accuracy and computational complexity. Methods for determining the most likely signal to which the observed feature set belongs are required by feature-based classification algorithms. Choosing the appropriate ML algorithm for signals classification is not the only challenge to overcome to obtain targeted classification accuracy; feature extractions and selection techniques are also important because they have a big impact on classification accuracy. Therefore, a reliable CFD detector is used for feature extraction in this work. In previous works, refs. [31,32] performed cyclostationary spectral analysis on the wideband spectrum resulting in high computational complexity; in order to reduce the overhead, this work exploits the sparse nature of WB, which is a valid assumption in the context of CR, and performed CFD analysis for the NB signal in each occupied sub-band in the WB spectrum. Further, a computationally efficient algorithm strip spectral correlation algorithm (SSCA) [33] is used to compute the SCF, as compared with the FFT accumulation method (FAM) [33] used in previous work. Further, a large dataset is collected using different carrier frequencies, jamming powers, and SNRs as compared with the jammer’s fixed power in [31,32]; here, varying jamming power can be considered as a mobile jammer scenario. In this work, a novel, single artificial neural network is proposed to detect and identify both multi-tone and pulse-modulated jammer attacks as well as to classify the legitimate signals with comparable accuracy to the work in [31,32].

While CR has emerged as a solution for DSA/OSA, its capacity to sense and explore a wide variety of frequencies and opportunistic applications has posed severe issues to network security. These features enable attackers to carry out more sophisticated attacks, for example, the primary user emulation (PUE) attacks [34], in which a hostile user impersonates a primary user. Furthermore, attackers can monitor the spectrum and use smart jamming to disrupt it [35,36]. Jamming attacks are Anomalous Spectrum Utilisation Attacks (ASUAs) that cause abnormal spectrum usage and disrupt the DSA/OSA in CR networks.

Our work considers stealthy jamming attacks. Such jammers are equipped with CR-spectrum sensing capability; therefore, they only transmit a jamming signal when activity is sensed over the channel and stop once the legitimate transmission stops. Such jammers are difficult to detect using common sensing techniques like ED at the physical layer. The majority of studies that investigate RF jamming assumed additive white Gaussian noise (AWGN) jamming [37,38].

However, the authors showed in [39,40] that modulation-based jamming attacks can result in optimum jamming in power-constrained conditions. We consider two different types of stealthy jammers: (i) the jammer is equipped with ED capabilities and uses a high-power multi-tone to jam various bands in WB radios; (ii) the jammer is equipped with a feature detector, able to identify the modulation schemes of a legitimate signal, and uses an optimum pulsed jamming strategy against the target signal. Hence, a reliable jammer detection algorithm is required to design a suitable anti-jamming system to counter such jamming attacks.

This article focuses on designing a reliable algorithm for the joint classification of legitimate signals and jammer detection in WB CRs. The main contributions of the article are the following:

1. Classify received licit signals into their corresponding modulation schemes using CFD and artificial neural network;
2. Detect both multi-tone and modulated pulsed stealthy jammers using the CFD and the same trained artificial neural network classifier as above.

We consider a WB spectrum that is constituted of many sub-bands, and each of the sub-bands is used by a narrowband (NB) signal or free. The occupied sub-bands are either used by a licit signal or jammed by the stealthy jammer. The algorithm’s first step is to perform cyclostationary spectral analysis on received NB signals and compute the corresponding SCF. Then, angular frequency profile (*f*) and cycle frequency profile (α) are obtained from the SCF of the signal. These two profiles are combined and used to train an artificial neural network, which then characterise various NB signals in a WB spectrum. Further, the algorithm is tested with an independent signal set at various SNRs. The algorithm has shown significantly high classification performances in the literature in comparison to the proposed techniques [41]. Moreover, it achieved very high jammer detection rates.

The article is structured as follows. Section 2 and Section 3 give the system model and proposed algorithm, respectively. Results are illustrated in Section 4. Finally, the conclusions are drawn in Section 5 with some future directions.

## 2. System Model and Problem Formulation

**Figure 1:**
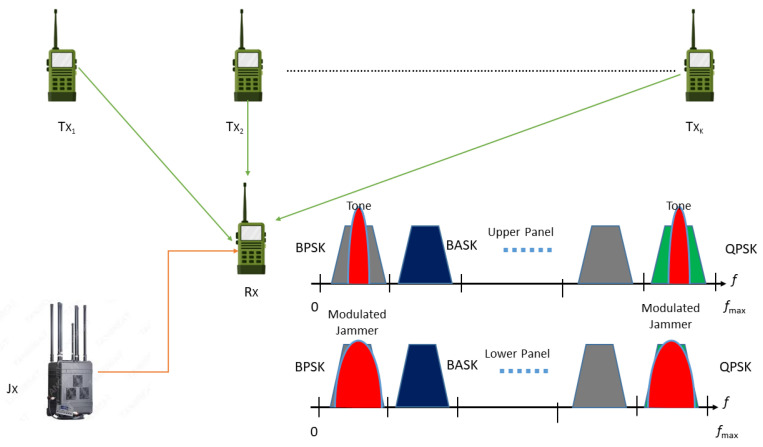
Cognitive radio terminal (Rx) senses a wideband spectrum composed of various narrowband transmitted signals. The multi-tone and modulated pulsed stealthy jammers try to jam different signals at the receiver node.

A number of transmitters ($Tx_{l}$), k s.t. l∈{0,1,2…M−1}, are present in the vicinity of CR terminal. The CR terminal sensed a WB spectrum and, therefore, received a signal that can be represented by the expression

(1) $$z\left(t\right)=\sum_{l=0}^{M-1}h_{l}\left(t\right)*S_{l}\left(t\right)+v\left(t\right)$$

where $S_{l}\left(t\right)$ shows the signal transmitted by the *l*-th transmitter, $h_{l}\left(t\right)$ is the radio channel between the *l*-th transmitter and receiver, ∗ represents convolution operator, and v(t) denotes the AWGN with zero mean and power spectral density of $\sigma_{v}^{2}$. We considered that the received NB signal’s power is degraded over the square of the distance; therefore, the free space path loss model (FSPL) is used to compute the received power at the receiver terminal. Further, it is considered that the channel ($h_{l}\left(t\right)$) is the slow, flat, Rayleigh fading channel in the observation process. It is assumed that transmitters can generate the signals with different modulation schemes, such as binary amplitude shift keying (BASK), binary phase shift keying (BPSK), quadrature amplitude modulation (QAM), quadrature phase shift keying (QPSK), pulse amplitude modulation (PAM), binary frequency shift keying (BFSK), or any other modulation scheme, as depicted in Figure 1. It shows that a receiver node scans a WB spectrum constituting multiple NB transmitted signals while a multi-tone jammer (upper panel) and modulated pulsed jammer (lower panel) try to jam multiple signals at the receiver terminal. The transmitters generate the signals using the following model:

(2) $$S_{l}{\left(t\right)}_{MPSK}=\sum_{n=-\infty }^{\infty }Ap\left(t-nT_{s}\right)e^{\left(j2\pi \left(m_{n}-1\right)/M+j2\pi f_{c}\right)}$$

where *A* is amplitude, $T_{s}=1/Rs$ is the symbol period and Rs is the symbol rate, M=2,4 are the number of unique phases, $m_{n}$ is the *n*-th transmitted symbol, $f_{c}$ is the carrier frequency, and p(t) is the Root Raised Cosine Pulse Shape (RCC) filter with a roll-off factor of β=0.5.

For our system model, two types of stealthy jammers are considered with different CR sensing capabilities:

1. Jammer is equipped with ED sensing technique and uses multi-tone as the jamming strategy to jam multiple NB signals in the observed WB signal. A tone with sufficiently higher power than the licit signal can jam any of the occupied SBs as shown in Figure 1.
2. Jammer is equipped with a feature detector; hence, it is able to recognise the modulation schemes of transmitted signals and, therefore, uses the optimal pulsed (modulated) jamming schemes against the target signals, as shown in Figure 1.

The pulsed jamming attacks are particularly effective in power-constrained environments, and optimal jamming schemes against modulated target signals are given in [40]. We assumed that both types of jammers are able to transmit powerful RF signals to cause interference at any communication frequency in the WB spectrum. Indeed, the received signal strength (RSS) related to the jamming signal depends on the distance between the jammer and the receiver terminal. Therefore, in order to simulate this scenario, the jammer-to-signal ratio (JSR) is fixed to 0 dB and the jammer terminal is moved towards receiver terminal from 15 m to 3 m with a step size of 3 m. In second scenario, the distance between the jammer and receiver terminals is fixed to 12 m and the JSR is changed between 0 dB and 7 dB. The dataset is collected for three broad jamming scenarios:

- No jamming: jammer is not transmitting
- Tone jamming: jammer employs multi-tone to jam the NB signals in WB spectrum
- Pulsed jamming: jammer employs pulsed jamming to jam the NB signals in WB spectrum. We used the MatLab environment to simulate the system model according to the specifications provided above.

## 3. Proposed Algorithm

This section first introduces cyclostationary spectral analysis and artificial neural networks; then, our newly proposed algorithm is presented.

### 3.1. Cyclostationary Spectral Analysis

The received signal z(t) is considered cyclostationary if its mean and autocorrelation function are periodic with period $T_{0}$,

(3) $$M_{z}\left(t+T_{0}\right)=M_{z}\left(t\right),\;R_{z}\left(t+T_{0},\tau \right)=R_{z}\left(t,\tau \right).$$

Fourier series components can be used to represent the autocorrelation of a cyclostationary signal z(t).

(4) $$R_{z}\left(t,\tau \right)=E\left[z\left(t+\tau /2\right)z^{*}\left(t+\tau /2\right)\right]$$

(5) $$R_{z}\left(t,\tau \right)=\sum_{\alpha }R_{z}^{\alpha }\left(\tau \right)e^{j2\pi \alpha t}$$

Here, E[.] is the expectation operator and $\alpha =\frac{b}{T_{0}}$, where *b* is an integer. $R_{z}^{\alpha }\left(\tau \right)$ is the cyclic autocorrelation function (CAF) of the received signal z(t) and given by the equation

(6) $$R_{z}\left(\tau \right)=\lim_{T\to \infty }\frac{1}{T}\int_{\frac{-T}{2}}^{\frac{T}{2}}R_{z}\left(t,\tau \right)e^{-j2\pi \alpha t}dt$$

as $R_{z}\left(\tau \right)$ is periodic with period $T_{0}$; therefore, (5) can be given by

(7) $$R_{z}\left(\tau \right)=\frac{1}{T_{0}}\int_{\frac{-T_{0}}{2}}^{\frac{T_{0}}{2}}R_{z}\left(t,\tau \right)e^{-j2\pi \alpha t}dt$$

The SCF is obtained by computing the Fourier Transform of the CAF (6) and given by

(8) $$S_{z}\left(f\right)=\int_{-\infty }^{\infty }R_{z}^{\alpha }\left(\tau \right)e^{-j2\pi f\tau }d\tau$$

where $S_{z}\left(f\right)$ is the SCF of received signal z(t), *f* and α represent the angular and cyclic frequencies, respectively.

**Figure 2:**
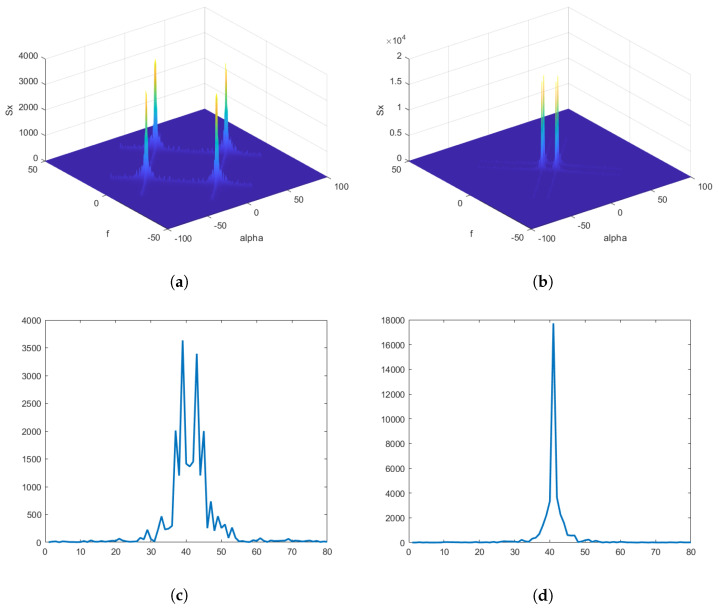
(**a**) SCF of BPSK signal. (**b**) SCF of QPSK signal. (**c**)Alpha profile of BPSK signal. (**d**) Alpha profile of QPSK signal.

The key advantage of using SCF is that its computation is not affected by noise, since noise is a stationary process and its spectral component has no correlation. This allows accurate computation of SCF even at very low SNRs. Moreover, modulated communications signals such as FSK, MSK, QAM, AM, PAM, QPSK, and BPSK with overlapped PSDs have unique SCF patterns. Since higher-order QAM and PSK modulation show the same second-order statistics, our experiments only considered BPSK and QPSK modulation schemes. Higher-order statistics [42] is needed for such signals that will be considered in the future to differentiate between higher-order QAM and PSK. The SCF of BPSK and QPSK signals is shown in Figure 2a,b. These panels show the spectral correlation densities of received NB signals as a function of both angular and cyclic frequencies. Since SCF estimation generates a large amount of data, it is not feasible to use it as a feature set for a classifier; therefore, two profiles—namely, *f*-profile and α-profile, given in Equations (9) and (10)—are obtained from SCF. The α-profile of NB BPSK and QPSK signal is depicted in Figure 2c,d. These profiles are combined to form an input feature vector that is then fed to an ANN-based classifier.

(9) $$I\left(\alpha \right)=max_{f}\left[S_{z}^{\alpha }\right]$$

(10) $$I\left(f\right)=max_{\alpha }\left[S_{z}^{\alpha }\right]$$

### 3.2. Artificial Neural Network and Proposed Algorithm

**Figure 3:**
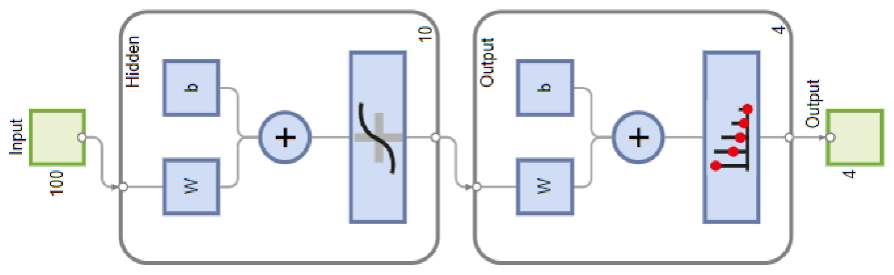
Proposed artificial neural network with ten neurons in hidden layer.

The proposed algorithm embeds an Artificial Neural Network (ANN) for spectrum characterisation because of its efficient use in pattern recognition problems. Further, it has the potential to generalise to any carrier frequency, signal-to-noise ratio, symbol rate, and frequency offset, which makes it suitable for the problem under consideration. The system is designed to characterise the spectrum under two stealthy jamming attacks, namely, multi-tone and modulated pulsed jamming attacks. First, a dedicated ANN is used as a classifier for each jamming attack. For multi-tone jamming attack, an ANN classifies received NB signals as BPKS, QPSK, BPSK plus Tone Jammer (BPSK-TJammed), and QPSK plus Tone Jammer (QPSK-TJammed). Similarly, for modulated pulsed jamming attacks, an ANN is used to characterise the signals as QPSK, BPSK, BPSK plus Pulsed Jammer (BPSK-PJammed), and QPSK plus Pulsed Jammer (QPSK-PJammed). ANN, like every supervised machine learning algorithm, operates in two stages: training (offline) and testing (online). The *f* and α profiles obtained from the SCF of each NB signal are concatenated and fed as a feature vector to ANN. Accordingly, both dedicated ANNs have 100 inputs associated with both the profiles, a hidden layer with ten neurons whose transfer function is a hyperbolic tangent sigmoid, and an output layer that contained four neurons associated with four signal classes, as discussed above. Each output value is between 0 and 1, and the class with the maximum value is treated as the signal type. The scale conjugate gradient back-propagation [43] algorithm is used to train ANN. For both dedicated ANN architectures, 100 trains are run, with weights being randomly initialised for each run. Each network architecture is trained (70%), validated (15%), and tested (15%) using a dataset of 40,000 signals. Over 98% (average) true positive classification was achieved with a single hidden layer for the four signal classes. Such classification accuracy indicates that an increase in the number of hidden layers will significantly increase the training time but not improve classification accuracy. As a result, the ANN architecture with a single hidden layer that consisted of 10 neurons and performed the best among the 100 trains was chosen for the classification of signals. Figure 3 depicts the ANN used in this work.

**Figure 4:**
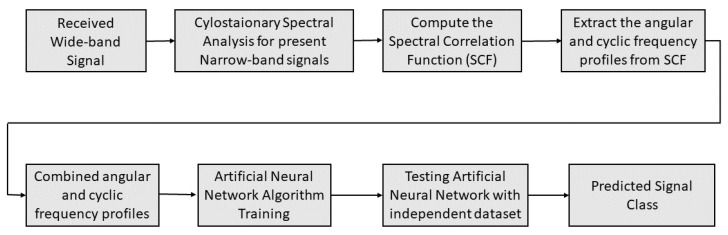
Proposed algorithm for joint signal classification and stealthy jammer detection.

Further, a single ANN is designed to classify the signals under both multi-tone and modulated pulsed stealthy jamming attacks. The ANN is aimed at classifying the signals as BPSK, QPSK, BPSK-TJammed, QPSK-TJammed, BPSK-PJammed, and QPSK-PJammed. The ANN is trained (70%), validated (15%), and tested (15%) using a dataset of 80,000 signals. For the classification of the above six classes of signals, the ANN with a single hidden layer that contained 18 neurons and demonstrated the best results among the 100 trains was chosen. The results are reported in Section 4. The block diagram of the proposed algorithm is depicted in Figure 4 and the pseudo-code of the algorithm is outlined in Algorithm 1. The CR terminal senses a WB spectrum that consists of many NB signals. Then, the SCF of each NB signal is computed according to the procedure detailed in Section 3.1, and *f*- and α-profiles are subsequently extracted from SCF. The *f*- and α-profiles of the respective NB signals are concatenated and given as input features to train the ANN. Then, the ANN is also tested for the independent signal set; it classifies the signal in occupied sub-bands as a legitimate signal with a corresponding modulation scheme or NB signal jammed by a particular type of jammer.
**Algorithm 1** Pseudo-code for proposed algorithm 1: **function** *Joint Signal Classification and Stealthy Jammer Detection* 2: **Input:** 3:     Train → Train ANN with Labelled data set 4:     Test → Independent data set 5: **Output:** 6:     Predicted → Signal class 7: **Procedure:** 8:     Initialise all SB states to “free” 9:     Receive the WB signal 10:    Divide WB into *j* SBs 11:    **for** j=1toJ, **do** 12:         Compute the SCF of each NB signal 13:         Obtain the α and *f*-profiles from SCF 14:         Feed the concatenated α and *f* frequency profiles for $SB_{j}$ to previously trained ANN 15:         Decision ← Signal class 16:    **end for** 17: **end function**

## 4. Simulation Results and Discussion

A WB signal of 50 MHZ, which is occupied by K NB signals, is considered to be sensed by a CR terminal. For our simulations in the MatLab environment, it is assumed that NB signals are generated by QPSK and BPSK modulation schemes. Two types of stealthy jamming attacks, namely, multi-tone and modulated pulsed, are considered against these signals. The WB spectrum is considered to be affected by Rayleigh fading and AWGN. The sampling rate is set to 100 MHz. It is assumed that detection is already performed and the CR node has knowledge of occupied SBs. The signal characterisation is performed at $\alpha =2f_{c}$, where AWGN has no correlation. The designed algorithm is further tested with the independent dataset and the classification results are given by confusion matrices.

### 4.1. Dedicated ANN Architecture for the Stealthy Jamming Attacks

A dedicated ANN architecture, for each type of jamming attack, is designed according to the process detailed in Section 3. The ANN is trained using a dataset of 40,000 signals at various carrier frequencies and SNRs; hence, the system performance is independent of carrier frequencies and SNRs.

**Figure 5:**
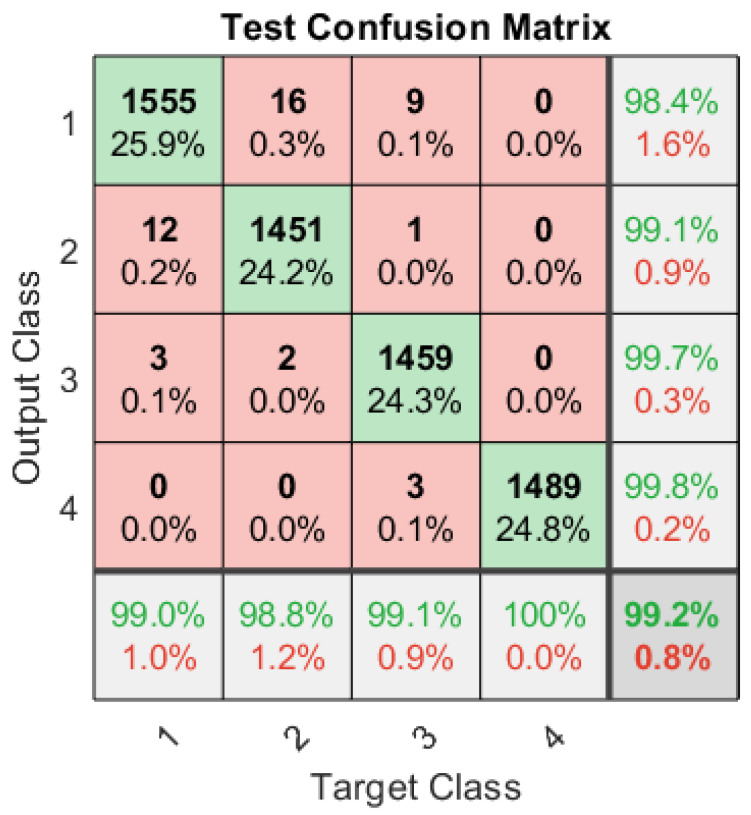
Test confusion matrix of the dedicated ANN with four output classes: BPSK, QPSK, BPSK-TJammed, and QPSK-TJammed.

The overall performance of the ANN classifier in the presence of a multi-tone stealthy jamming attack is shown in the form of a test confusion matrix in Figure 5 that shows a classification rate of approximately 99% is achieved. The tested signals are 15% (6000) of the total signals used to train ANN. Figure 5 shows that the algorithm successfully identified the un-jammed legitimate signals, QPSK as QPSK and BPSK as BPSK, with 99% and 98% accuracy, respectively. For both jammed signals, BPSK-TJammed and QPSK-TJammed, the classification accuracy is approximately 99.7% and 99.8%. After training and testing the ANN with 40,000 samples, the ANN is further tested using an independent signal set. The system’s performance is specifically evaluated online for 1000 independent signals that are generated using various carrier frequencies and SNRs (−9 to 6 dB). The confusion matrices in Table 1, Table 2, Table 3, Table 4, Table 5 and Table 6 give the classification rate for the four classes of signals. The proposed algorithm, which is based on cyclic spectral analysis and ANN, performs well in most of the system configurations, even at low SNRs. It can be observed in Table 1 that at −9 dB, the ANN classified BPSK and QPSK to their corresponding classes with a rate of 97.7% and 96.7%, while for 98.7% and 99.2%, the algorithm correctly classified the jammed signals (BPSK-Jammed and QPSK-Jammed). Table 1 shows that for all four classes of signals, a classification rate of approximately 99% is achieved. Further, it is possible to infer from confusion matrices (Table 2, Table 3, Table 4, Table 5, Table 6 and Table 7) that no classification errors (100% accuracy) are observed at and above −3 dB.

**Figure 6:**
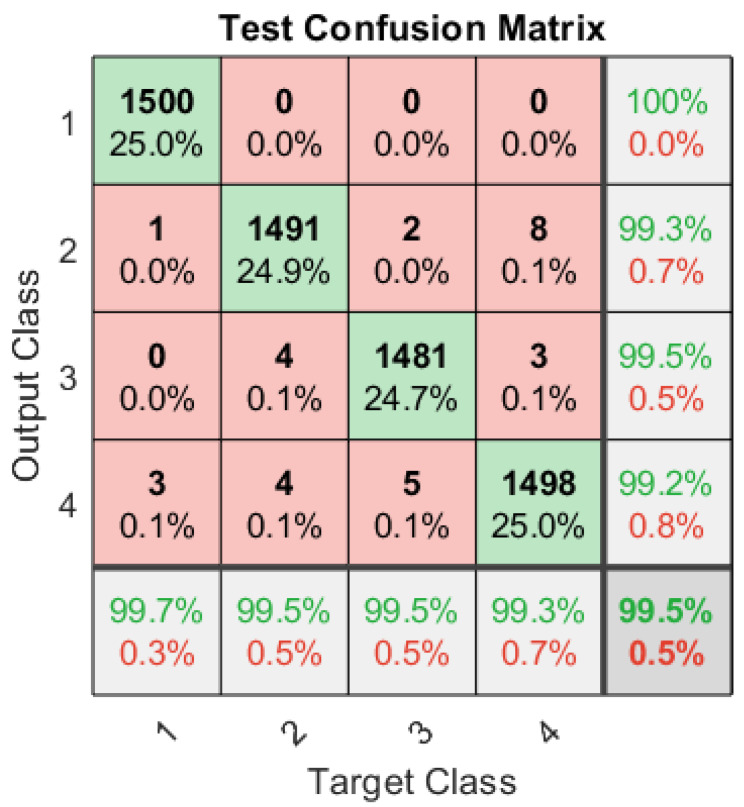
Test confusion matrix of the dedicated ANN with four output classes: BPSK, QPSK, BPSK-PJammed, and QPSK-PJammed.

Similarly, a dedicated ANN is trained for a total 40,000 signals set at different SNRs and carrier frequencies to classify the signals in the presence of modulated pulsed jamming attacks. ANN classifies the signals into four classes, namely, BPSK, QPSK, BPSK-PJammed, and QPSK-PJammed. The optimal jamming strategies for such a jammer are given in Table 1. The test confusion matrix for signal classification under modulated pulsed stealthy jamming attack is given in Figure 6 and the results show that the proposed ANN correctly classifies all signals with an overall classification of 99.5%. Moreover, the ANN is further tested for an independent signal set and test confusion matrices for various SNRs are shown in Table 7, Table 8, Table 9, Table 10, Table 11 and Table 12. Table 7 shows that at −9 dB, for all four types of signals—BPSK, QPSK, BPSK-PJammed, and QPSK-Jammed—the classification rate is approximately 99%. Table 9, Table 10, Table 11 and Table 12 show that a classification rate of 100% is achieved at and above −3 dB, which makes this algorithm suitable to not only classify the legitimate signals but also detect jamming attacks at low SNRs.

### 4.2. A Single ANN Architecture for Both Stealthy Jamming Attacks

**Figure 7:**
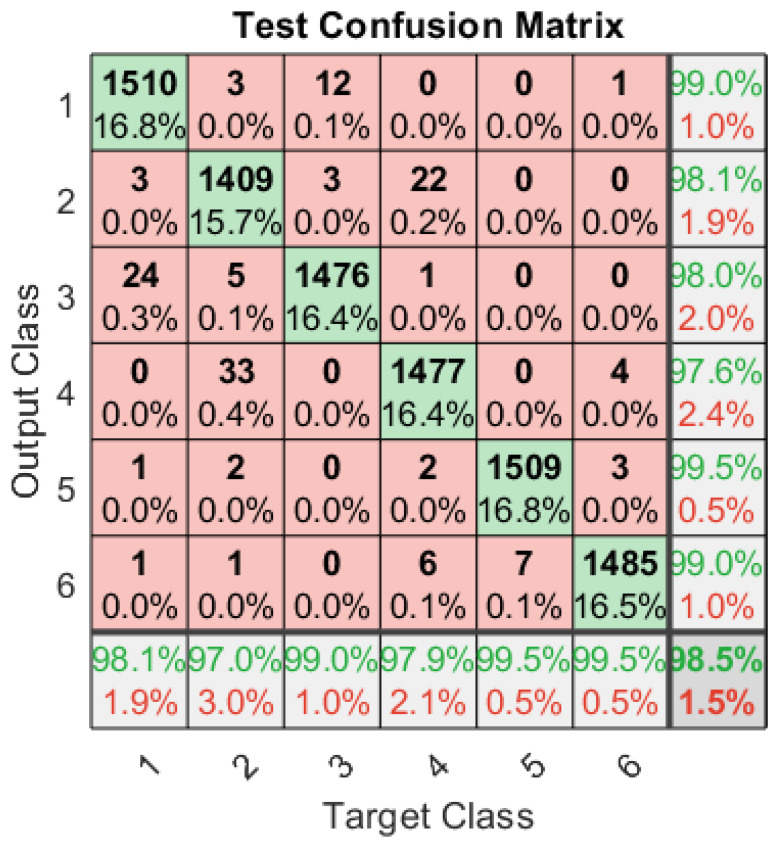
Confusion matrix of the single ANN architecture with six output classes: BPSK, QPSK, BPSK-PJammed, QPSK-PJammed, BPSK-TJammed, and QPSK-TJammed.

A single ANN is designed to characterise the spectrum in the presence of both multi-tone and modulated pulsed jamming attacks. The ANN is trained with 80,000 signals set at different SNRs and carrier frequencies for six classes of signals, which are BPSK, QPSK, BPSK-Modulated Pulse Jammed (BPSK-PJ), QPSK-Modulated Pulse Jammed (QPSK-PJ), BPSK-Tone Jammed (BPSK-TJ), and QPSK-Tone Jammed (QPSK-TJ). The ANN classifies the signals with a total classification rate of 98.5%, as presented in Figure 7. The true positive for six classes are 99%, 98%, 98%, 97%, 99%, and 99% consecutively, which shows that the proposed algorithm not only classifies the legitimate signals with a very high rate but also detects both multi-tone and modulated pulsed stealthy jamming attacks with very high accuracy. Further, this single ANN gives comparable performance to dedicated ANN architectures (Figure 5 and Figure 6) that used a dedicated neural network to detect one particular type of jamming attack.

Moreover, the ANN is also tested for independent signals from the one used in the previous confusion matrix computation. For testing, 1000 samples, for each class of signals, are tested at SNRs in the range of −9–6 dB. The resultant confusion matrices for each SNR are shown in Table 13, Table 14, Table 15, Table 16, Table 17 and Table 18. The results show that at −9 dB, the classification rates for un-jammed signals, BPSK and QPSK, are 96.8% and 91.4%, while for four jammed signals—BPSK-PJ, QPSK-PJ, BPSK-TJ, and QPSK-TJ—they are 90.7%, 95.4%, 93.3%, and 96%, respectively. Classification performance is reduced compared with dedicated ANN architecture, for example, the classification rate at −9 dB is approximately 93% compared with 98% with dedicated ANN (Table 2 and Table 8) architecture. Similarly, the detection rate of the jammer is also reduced to 93% compared with 99% with dedicated ANN for each jamming attack. However, the performance of the algorithm is increased at −6 dB and classified the first four classes (BPSK, QPSK, BPSK-PJ, and QPSK-PJ) with a rate of approximately 98%, whereas the last two classes of signals (BPSK-TJ and QPSK-TJ) are classified with a rate of 99.5%. A similar trend is observed at −6 dB and a classification rate in dedicated ANN is approximately 100%, whereas it is 97% for single ANN architecture.

**Figure 8:**
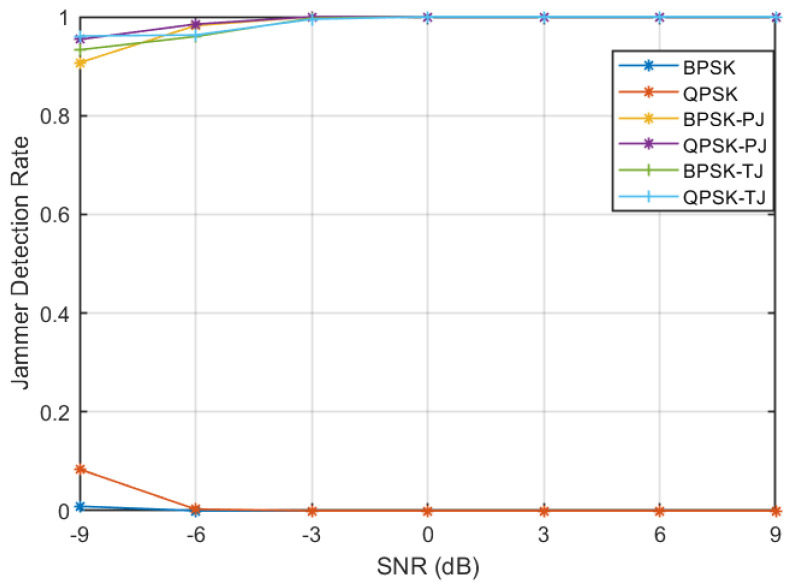
Jammer detection rate at various SNRs.

**Figure 9:**
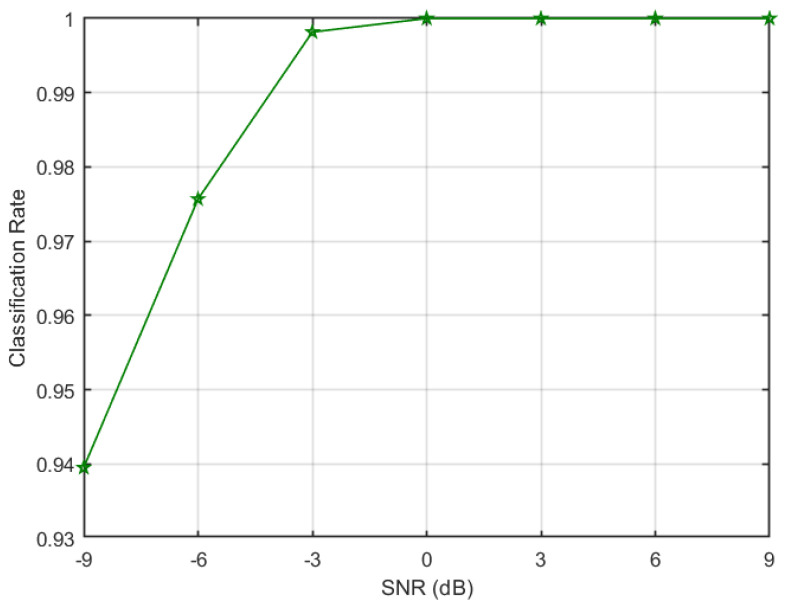
Signal’s classification rate at various SNRs.

A total classification rate of approximately 100% is achieved at −3 dB, which means ANN is able to correctly classify all the signals without any errors. This performance is comparable to dedicated ANN architecture; therefore, it can be inferred from the results that a single ANN architecture can be selected to detect both types of stealthy jamming attacks as well as to classify legitimate signals to their corresponding modulation schemes. The jammer detection rate achieved by the algorithm at different SNRs is plotted in Figure 8. It can be noticed from the figure that a very high jammer detection rate of 0.97 is achieved at low SNR of −6 dB, whereas a jammer detection rate of 1 is attained at −3 dB. Moreover, it can be seen that a very low miss-classification rate of legitimate signals as a jammer is obtained. Further, to evaluate the robustness of the algorithm, the overall signal correct classification rate ordained at various SNRs is given in Figure 9. The figure shows that a classification rate of 0.975 is achieved at −6 dB and increased to 1 at −3 dB. The algorithm achieved such high performances because the SCF of different types of signals results in highly distinct patterns; therefore, corresponding α profiles of signals can be used as a feature set (refer to Section 3.1). Further, an ANN-based classifier is shown to be a robust tool to recognise such patterns of various signals at low SNRs.

Further, our proposed algorithm achieved significantly high accuracy when compared with most recent techniques for signal classifications that require 10–20 dB SNR for comparable classification performances [44,45,46,47,48]. For example, in [49], the authors presented an algorithm based on instantaneous statistical characteristics and a Support Vector Machine (SVM) capable of classifying modulated signals 2ASK, 4ASK, 2FSK, and 2PSK with a classification rate of 0.95 at 5 dB and can only attain error-free classification at approximately 14 dB. On the other hand, our proposed algorithm achieved a classification rate of 0.975 even at −6 dB, showing a significant performance gain due to the use of expert features that are extracted from signals using cyclostationary spectral analysis. On the other hand, our proposed algorithm achieved a classification rate of 0.975 even at −6 dB, resulting a significant performance gain due to the use of expert features that are extracted from signals using cyclostationary spectral analysis. In [45], the jammer detection algorithm uses the centre frequencies, the peak amplitude of the PSD, and bandwidths of the NB signals as the feature set for the naive Bayes Classifier (NBC). The result shows that at 5 dB the algorithm is able to classify the un-jammed signals (BPSK and QPSK) and jammed signals (BPSK-Jammed and QPSK-Jammed) at a rate of 0.82 and 0.69 (without compression), respectively. Similarly, a classification rate of 0.88 and 0.79 is achieved at 10 dB. It can be observed from the results shown in Table 15 that our proposed technique outperformed the algorithm in [45] and achieved a classification rate of 0.98 for un-jammed and 0.97 at −6 dB. Further, our designed algorithm achieved error-free classification even at −3 dB. Further, it can be noticed that the newly proposed single ANN architecture yields approximately the same accuracy when validated over a large dataset of 80,000 samples, in comparison to our previous work in [31,32], which used a dedicated ANN architecture to detect each type of jamming attack. Such validations show that a single ANN-based algorithm is highly reliable and robust.

## 5. Conclusions

In this article, a novel algorithm is presented for joint signal classification and stealthy jammer detection in WB radios. The WB is composed of numerous NB signals that could be either licit signals or jammed by a stealthy jammer. The jammers embed energy detection and feature detection capabilities of CR and use multi-tone and modulated pulsed jamming signals to jam the licit NB signals. The received NB signals in WB are fed to the CFD module that computes the corresponding SCF. The features related to α and *f* profiles are obtained from SCF; then, they are concatenated and provided as input features to the ANN that classifies the signals either as legitimate signals with corresponding modulation schemes or jammers with a particular jamming attack (multi-tone and modulated pulsed). The performances of both the ANN-based classifiers, dedicated networks and single network, are evaluated at different SNRs and classification results are given by confusion matrices. The results showed that our newly proposed algorithm performed well at low SNRs (−6–0 dB) and, even with single ANN architecture, a classification accuracy of 0.98 is achieved at −6 dB. Further, the designed algorithm showed superior performances when compared with recently proposed signal classifications and jammer detection algorithms.

Future work will include the development of an autonomous system capable of dynamically accessing the WB spectrum in a CR environment for applications like defence against more sophisticated jamming attacks. Another interesting future research direction could be exploring deep learning for signal classification that does not need expert features.

## Figures and Tables

**Table 1:** Confusion matrix of the proposed ANN at −9 dBin presence of Tone jamming.

Signal Class	BPSK	QPSK	BPSK-TJammed	QPSK-TJammed
BPSK	977	21	2	0
QPSK	33	967	0	0
BPSK-TJammed	2	3	987	8
QPSK-TJammed	0	0	8	992

**Table 2:** Confusion matrix of the proposed ANN at −6 dB in presence of Tone jamming.

Signal Class	BPSK	QPSK	BPSK-TJammed	QPSK-TJammed
BPSK	990	10	0	0
QPSK	13	985	0	3
BPSK-TJammed	0	0	992	8
QPSK-TJammed	0	0	2	998

**Table 3:** Confusion matrix of the proposed ANN at −3 dBin presence of Tone jamming.

Signal Class	BPSK	QPSK	BPSK-TJammed	QPSK-TJammed
BPSK	1000	0	0	0
QPSK	0	1000	0	0
BPSK-TJammed	0	0	1000	0
QPSK-TJammed	0	0	0	1000

**Table 4:** Confusion matrix of the proposed ANN at 0 dBin presence of Tone jamming.

Signal Class	BPSK	QPSK	BPSK-TJammed	QPSK-TJammed
BPSK	1000	0	0	0
QPSK	0	1000	0	0
BPSK-TJammed	0	0	1000	0
QPSK-TJammed	0	0	0	1000

**Table 5:** Confusion matrix of the proposed ANN at 3 dBin presence of Tone jamming.

Signal Class	BPSK	QPSK	BPSK-TJammed	QPSK-TJammed
BPSK	1000	0	0	0
QPSK	0	1000	0	0
BPSK-TJammed	0	0	1000	0
QPSK-TJammed	0	0	0	1000

**Table 6:** Confusion matrix of the proposed ANN at 6 dBin presence of Tone jamming.

Signal Class	BPSK	QPSK	BPSK-TJammed	QPSK-TJammed
BPSK	1000	0	0	0
QPSK	0	1000	0	0
BPSK-TJammed	0	0	1000	0
QPSK-TJammed	0	0	0	1000

**Table 7:** Confusion matrix of the proposed ANN at −9 dB in presence of Pulsed jamming.

Signal Class	BPSK	QPSK	BPSK-PJammed	QPSK-PJammed
BPSK	990	5	3	2
QPSK	3	988	2	7
BPSK-PJammed	0	0	992	8
QPSK-PJammed	0	0	5	995

**Table 8:** Confusion matrix of the proposed ANN at −6 dBin presence of Pulsed jamming.

Signal Class	BPSK	QPSK	BPSK-PJammed	QPSK-PJammed
BPSK	990	10	0	0
QPSK	13	985	0	3
BPSK-PJammed	0	0	992	8
QPSK-PJammed	0	0	2	998

**Table 9:** Confusion matrix of the proposed ANN at −3 dBin presence of Pulsed jamming.

Signal Class	BPSK	QPSK	BPSK-PJammed	QPSK-PJammed
BPSK	1000	0	0	0
QPSK	0	1000	0	0
BPSK-PJammed	0	0	1000	0
QPSK-PJammed	0	0	0	1000

**Table 10:** Confusion matrix of the proposed ANN at 0 dBin presence of Pulsed jamming.

Signal Class	BPSK	QPSK	BPSK-PJammed	QPSK-PJammed
BPSK	1000	0	0	0
QPSK	0	1000	0	0
BPSK-PJammed	0	0	1000	0
QPSK-PJammed	0	0	0	1000

**Table 11:** Confusion matrix of the proposed ANN at 3 dBin presence of Pulsed jamming.

Signal Class	BPSK	QPSK	BPSK-PJammed	QPSK-PJammed
BPSK	1000	0	0	0
QPSK	0	1000	0	0
BPSK-PJammed	0	0	1000	0
QPSK-PJammed	0	0	0	1000

**Table 12:** Confusion matrix of the proposed ANN at 6 dBin presence of Pulsed jamming.

Signal Class	BPSK	QPSK	BPSK-PJammed	QPSK-PJammed
BPSK	1000	0	0	0
QPSK	0	1000	0	0
BPSK-PJammed	0	0	1000	0
QPSK-PJammed	0	0	0	1000

**Table 13:** Confusion matrix of the proposed ANN at −9 dB in presence of Tone and Pulsed jamming.

Signal Class	BPSK	QPSK	BPSK-PJ	QPSK-PJ	BPSK-TJ	QPSK-TJ
BPSK	968	23	9	0	0	0
QPSK	2	914	15	1	1	67
BPSK-PJ	9	7	907	1	71	5
QPSK-PJ	0	0	2	954	1	43
BPSK-TJ	0	0	48	5	933	14
QPSK-TJ	0	3	0	23	13	961

**Table 14:** Confusion matrix of the proposed ANN at −6 dB in presence of Tone and Pulsed jamming.

Signal Class	BPSK	QPSK	BPSK-PJ	QPSK-PJ	BPSK-TJ	QPSK-TJ
BPSK	980	20	0	0	0	0
QPSK	13	984	3	0	0	0
BPSK-PJ	0	1	982	10	7	0
QPSK-PJ	0	0	0	985	1	14
BPSK-TJ	0	0	35	5	960	0
QPSK-TJ	0	9	0	25	3	963

**Table 15:** Confusion matrix of the proposed ANN at −3 dBin presence of Tone and Pulsed jamming.

Signal Class	BPSK	QPSK	BPSK-PJ	QPSK-PJ	BPSK-TJ	QPSK-TJ
BPSK	999	1	0	0	0	0
QPSK	2	998	0	0	0	0
BPSK-PJ	0	0	1000	0	0	0
QPSK-PJ	0	0	0	1000	0	0
BPSK-TJ	0	0	0	3	997	0
QPSK-TJ	0	0	0	5	0	995

**Table 16:** Confusion matrix of the proposed ANN at 0 dBin presence of Tone and Pulsed jamming.

Signal Class	BPSK	QPSK	BPSK-PJ	QPSK-PJ	BPSK-TJ	QPSK-TJ
BPSK	1000	0	0	0	0	0
QPSK	0	1000	0	0	0	0
BPSK-PJ	0	0	1000	0	0	0
QPSK-PJ	0	0	0	1000	0	0
BPSK-TJ	0	0	0	0	1000	0
QPSK-TJ	0	0	0	0	0	1000

**Table 17:** Confusion matrix of the proposed ANN at 3 dBin presence of Tone and Pulsed jamming.

Signal Class	BPSK	QPSK	BPSK-PJ	QPSK-PJ	BPSK-TJ	QPSK-TJ
BPSK	1000	0	0	0	0	0
QPSK	0	1000	0	0	0	0
BPSK-PJ	0	0	1000	0	0	0
QPSK-PJ	0	0	0	1000	0	0
BPSK-TJ	0	0	0	0	1000	0
QPSK-TJ	0	0	0	0	0	1000

**Table 18:** Confusion matrix of the proposed ANN at 6 dBin presence of Tone and Pulsed jamming.

Signal Class	BPSK	QPSK	BPSK-PJ	QPSK-PJ	BPSK-TJ	QPSK-TJ
BPSK	1000	0	0	0	0	0
QPSK	0	1000	0	0	0	0
BPSK-PJ	0	0	1000	0	0	0
QPSK-PJ	0	0	0	1000	0	0
BPSK-TJ	0	0	0	0	1000	0
QPSK-TJ	0	0	0	0	0	1000

## Data Availability

The data used to support the findings of this study are available from the corresponding author upon request.
